# BraTioUS: A multicenter dataset of baseline intraoperative brain tumor ultrasound images

**DOI:** 10.1016/j.dib.2026.112478

**Published:** 2026-01-15

**Authors:** Olga Esteban-Sinovas, Rosario Sarabia, Ignacio Arrese, Vikas Singh, Prakash Shett, Aliasgar Moiyadi, Ilyess Zemmoura, Massimiliano Del Bene, Arianna Barbotti, Francesco DiMeco, Timothy Richard West, Brian Vala Nahed, Giuseppe Roberto Giammalva, Santiago Cepeda

**Affiliations:** a Department of Neurosurgery, Río Hortega University Hospital, Valladolid 47014, Spain; b Specialized Group in Biomedical Imaging and Computational Analysis (GEIBAC), Instituto de Investigacion Biosanitaria de Valladolid (IBioVALL), Valladolid 47014, Spain; c Department of Neurosurgery, Tata Memorial Centre, Homi Bhabha National Institute, Mumbai, Maharashtra 400012, India; d UMR 1253, iBrain, Université de Tours, Inserm, Tours 37000, France; e Department of Neurosurgery, CHRU de Tours, Tours 37000, France; f Department of Neurosurgery, Fondazione IRCCS Istituto Neurologico Carlo Besta, Via Celoria 11, Milan 20133, Italy; g Department of Pharmacological and Biomolecular Sciences, University of Milan, Milan 20122, Italy; h Department of Oncology and Hematology-Oncology, Università Degli Studi di Milano, Milan 20122, Italy; i Department of Neurological Surgery, Johns Hopkins Medical School, Baltimore, MD 21205, USA; j Department of Neurosurgery, Massachusetts General Hospital, Mass General Brigham, Harvard Medical School, Boston, MA 02114, USA; k Neurosurgery Department, ARNAS Civico Di Cristina Benfratelli Hospital, Palermo 90127, Italy

**Keywords:** Ultrasonography, Brain tumor, Glioma, Deep-learning, Segmentation

## Abstract

The BraTioUS (Brain Tumor Intraoperative Ultrasound) dataset [1] is a large-scale, multicenter, and publicly available collection of intraoperative ultrasound (ioUS) images acquired during glioma surgeries. Created through an international collaboration among six hospitals across five countries, BraTioUS comprises 1669 B-mode 2D ioUS images from 142 glioma patients collected between 2018 and 2023 using various ultrasound systems and acquisition protocols. It also includes masks supervised by experts of tumor segmentation from every ioUS image.

BraTioUS addresses several limitations found in existing public datasets, such as lack of diversity in acquisition hardware, imaging protocols, and glioma types. The primary objective of this dataset is to be publicly available and accessible for the training and validation of machine learning models aimed at improving the interpretation and use of ioUS. The dataset’s scale, quality, and heterogeneity make it a valuable resource for training and validating AI tools aimed at improving intraoperative decision-making and patient outcomes in glioma surgery.

Specifications Table

**Table:** 

Subject	Health Sciences, Medical Sciences & Pharmacology
Specific subject area	Intraoperative ultrasound imaging of brain gliomas for artificial intelligence model development, segmentation, and surgical guidance.
Type of data	Intraoperative Brain Ultrasound Images, Processed images, Segmentation masks
Data collection	B-mode intraoperative ultrasound images were acquired during glioma resections performed between 2018 and 2023 at six neurosurgical centers (Rio Hortega University Hospital, Valladolid, Spain (RHUH); Tata Memorial Center, Mumbai, India (TMC); Le Centre Hospitalier Régional Universitaire de Tours, France (CHRUT); Carlo Besta Neurological Institute, Milan, Italy (INCC); University of Palermo, Italy (UPALER); and Massachusetts General Hospital, Boston, USA (MGH). Ultrasound images were acquired using sterilizable neuro-cranial probes or probes covered with sterile sheets on cart-based ultrasound systems from various manufacturers and models (Hitachi©, Esaote©, BK©, Supersonic© and Sonowand©). Probes used to obtain ioUS images were linear or curve according to manufacturer. Image acquisition planes were obtained as closely as possible to the conventional orthogonal planes, subject to craniotomy constraints. Images with suboptimal quality or artifacts impeding interpretation were excluded. All data were anonymized and standardized removing acquisition-related and patient-identifying elements. BraTioUS Database includes segmentation masks of tumor tissue that appears in 1669 images, which was performed using a previous configurated nnU-Net model [2]. All pseudolabels automatically created were manually reviewed and corrected by neurosurgeons specialized in interpretation and analysis of intraoperative ultrasound. Manual segmentation was performed using ITK-SNAP software (Version 4.0.1, http://itksnap.org, accessed on 1 November 2024). Final datasets comprise 1669 NIfTI-format 2D ioUS images and 1669 manual expert refined tumor labels.
Data source location	Institution: Rio Hortega University Hospital City/Town/Region: Valladolid, Castile and Leon Country: Spain
Data accessibility	Repository name: BraTioUS - Brain Tumor Intraoperative Ultrasound Dataset Data identification number: 10.5281/zenodo.16887362 Direct URL to data: https://zenodo.org/records/16887363
Related research article	Cepeda, S.; Esteban-Sinovas, O.; Singh, V.; Shetty, P.; Moiyadi, A.; Dixon, L.; Weld, A.; Anichini, G.; Giannarou, S.; Camp, S.; et al. Deep Learning-Based Glioma Segmentation of 2D Intraoperative Ultrasound Images: A Multicenter Study Using the Brain Tumor Intraoperative Ultrasound Database (BraTioUS). Cancers 2025, 17, 315. https://doi.org/10.3390/cancers17020315 [2]

## 1 Value of the Data

1. Multicenter collaboration: For executing this project, six independent hospital centers from five different countries agreed to create and organize a publicly shared database of intraoperative ultrasound images of brain tumor surgeries. BraTioUS Consortium (ClinicalTrials.gov Identifier: NCT0562772) comprises contributions from the following hospital centers:
  - Rio Hortega University Hospital, Valladolid, Spain (RHUH): 58 patients, 738 images;
  - Tata Memorial Center, Mumbai, India (TMC): 35 patients, 666 images;
  - Le Centre Hospitalier Régional Universitaire de Tours, France (CHRUT): 29 patients, 177 images;
  - Carlo Besta Neurological Institute, Milan, Italy (INCC): 10 patients, 55 images;
  - Massachusetts General Hospital, Boston, USA (MGH): 5 patients, 19 images.
  - University of Palermo, Italy (UPALER): 5 patients, 14 images;
2. Dataset size and variability: To the best of our knowledge, this is the largest publicly available database of intraoperative ultrasound (ioUS) data for brain tumor surgery, both in the number of images and cases, as well as in the inclusion of tumor segmentations. It contains 1669 images from 142 patients who underwent glioma resection. Multicenter data collection encompasses multiple scanner models, manufacturers, and acquisition protocol, which introduces a level of variability absent in existing public datasets (BITE [3], RESECT [4], ReMIND [5], which are typically limited in geographical distribution, equipment and technological heterogeneity. Such variability enhances the dataset’s representativeness, supporting the development and validation of artificial intelligence (AI) and machine learning (ML) [6] models with improved generalizability and robustness for clinical applications, including tumor detection, segmentation, surgical navigation, and outcome prediction [[7], [8], [9]]. Table 1 provides a comparative overview of the characteristics of the different databases of oiUS.

**Table 1:** Comparative features of the four currently publicly accessible datasets.

	NMT-BITE	RESECT	ReMIND	BraTioUS
**Group**	Montreal	Trondheim	Boston	Multicenter (RHUH, TMC, CHRUT, INCC, UPALER, MGH)
**Publication Year**	2012, 2014, 2016	2017, 2022	2023	Present
**N° Patients**	14	23	114	142
**N° US images**	89	69	320	1669
**Tumor types**	Low- Grade and High- Grade Gliomas	Low-grade	Low-Grade, High-Grade Gliomas and other pathology	High Grade and Low Grade Gliomas
**MR images**	Yes (pre- and postoperative)	Yes (preoperative)	Yes (pre-, intra- and postoperative)	No
**ioUS manufacturer**	Philips ATL	Sonowand	BK medical	Variable (Hitachi, BK, Sonowand, Supersonic, Esaote)
**ioUS timepoint**	Pre-resection and post-resection	Extradural, resection control and postresection	Pre-resection (extra and intradural) and post-resection	Pre-resection (intra and extradural)
**Acquisition type**	2D & 3D	3D	3D	2D
**Frequency**	4-7 MHz	12-6 Mhz	13-5 Mhz	Variable
**Probe**	-	Linear	Curved	Linear/Curved
**Type of image segmented**	MR	MR & ioUS	MR	ioUS
**Segmentation mode**	Manual (preop. tumor)	Manual (preop. tumor and resection cavity)	- Manual (preop. tumor, prior resection cavity) - Automatic (preop. ventricles, preop cerebrum)	Automatic + expert correction (preop tumor)
3. Bidimensional vs. volumetric images: ioUS images available are two-dimensional (2D) rather than volumetric presentation, offering enhanced image resolution and quality, which supports more efficient processing and effective training of artificial intelligence algorithms. Other related datasets consist solely of three-dimensional acquisitions (3D), which frequently require conversion into 2D slices for further analysis. This conversion can compromise the quality of segmentation, as many slices may not contain relevant tumor features, and the resolution of 3D images is typically lower than that of native 2D acquisitions.
4. High-grade tumors: In this dataset, a total of 88.53% of the resected tumors are high-grade gliomas, providing the sample with homogeneity in terms of pathology and the prognostic significance associated with the extent of tumor resection. The other ioUS images datasets are largely limited to cases involving low-grade gliomas, some even include non-tumoral pathology.
5. Fully anonymized for external use: The images were subjected anonymization procedures employing an automatic segmentation model based on nn-UNet, which systematically removed extraneous elements related to data acquisition, patient identifiers, and other non-essential information. These steps ensured that the dataset is compliant with standards for external use. The main aim of the project is to provide open access to ioUS images of brain tumors, thereby facilitating the development and validation of artificial intelligence–driven tools for intraoperative decision support and biomedical research in brain tumor surgery. As referenced previously, it has already been employed in publications by this research group with excellent results, both in prognostic estimation [7] and in real-time image detection [8].
6. Tumor segmentations supervised by experts and manually corrected: ioUS constitutes a valuable tool for the identification of tumor tissue during brain tumor resection. However, the intrinsic characteristics of ultrasound imaging often make this distinction challenging, due to artifacts, low resolution, or limited contrast between healthy and pathological tissue. By providing 1669 tumor tissue segmentations from the corresponding ioUS images, the aim is to support new studies focused on training and validating segmentation models in ultrasound. The following sections describe in detail the segmentation process and how the 1669 labels were reviewed and corrected to optimize the dataset.

## 2 Background

Intraoperative imaging plays a pivotal role in brain tumor surgery, particularly for gliomas, which account for 30% of primary brain tumors and 80% of malignant cases [6]. The extent of resection is a critical prognostic factor, yet achieving maximal safe resection remains challenging due to the infiltrative nature of gliomas and their proximity to eloquent brain regions [10]. Intraoperative ultrasound (ioUS) provides real-time imaging with notable advantages over intraoperative MRI, including portability, cost-effectiveness, and seamless integration into the surgical workflow [11,12]. However, its broader adoption is hindered by operator dependency, a steep learning curve, and interpretation challenges caused by artifacts, low contrast between tumor and healthy tissue, non-orthogonal planes and a limited field of view [13,14].

AI and ML models are being developed to enhance navigation, segmentation, prognosis, and recurrence prediction in neuro-oncology and specifically, in intraoperative imaging [7,8,[15], [16], [17], [18]]. Clinical implementation of these models requires large, diverse datasets. Nevertheless, only three ioUS images of brain tumor datasets have been public available: MNI BITE [3], RESECT [4], and ReMIND [5]. These are valuable resources but are limited by diversity, resolution, acquisition modality, and tumor type representation, underscoring the need for expanded and higher-quality datasets to advance AI-assisted ioUS applications in neurosurgery.

## 3 Data Description

The BraTioUS database is organized into threecompressed folders in ZIP format. The first folder contains the 1669 intraoperative ultrasound (ioUS) images that constitute the dataset, stored in Neuroimaging Informatics Technology Initiative (NIfTI) format (.nii). Each file is labeled with a numerical code identifying the patient, followed by a second number corresponding to the specific image (e.g., “BraTioUS-001-04”, where “001” refers to the subject and “04” to the image number for that subject). These archives can be extracted using standard decompression utilities available in most operating systems (e.g., unzip in Unix-based environments, built-in extraction tools in Windows and macOS) or widely used open-source software such as 7-Zip or WinRAR. Upon decompression, the original NIfTI files retain their full spatial resolution and metadata, enabling direct use in neuroimaging analysis pipelines.

The second ZIP folder contains NIfTI-format segmentations of the tumor lesions depicted in the corresponding ultrasound images. The extraction process for these files would be the same as previously described .The file naming follows the same structure as in the first folder. NIfTI files can be opened and inspected using widely adopted neuroimaging software packages such as FSLeyes (FSL), MRIcron, ITK-SNAP, 3D Slicer, or Freeview (FreeSurfer).

**Fig. 1:**
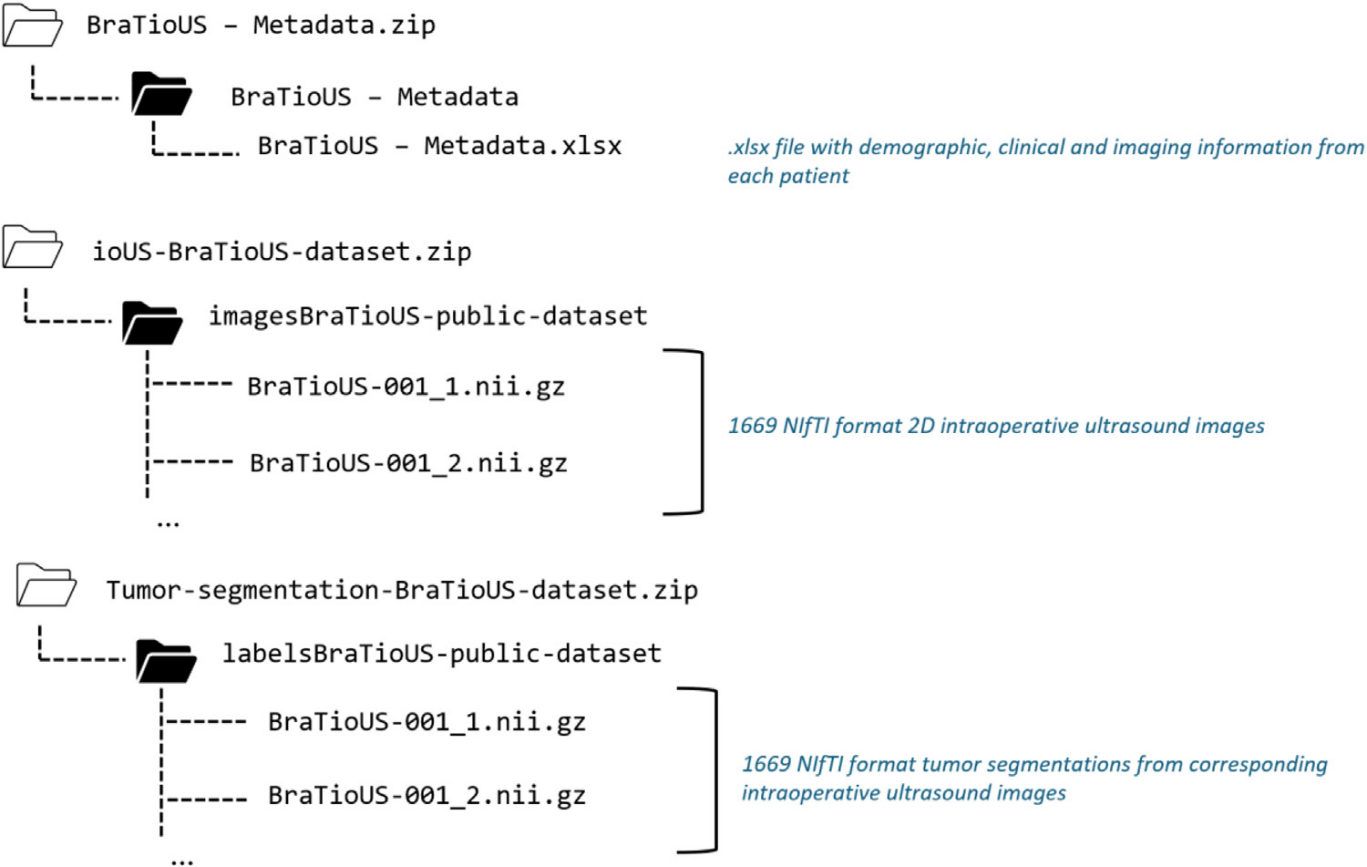
Schematic representation of the directory folder and file structure.

The repository also includes a third ZIP folder that includes a .xlsx file (Microsoft® Excel®) containing anonymized metadata for the dataset, comprising demographic, clinical, and imaging information for each subject (Fig. 1).

## 4 Experimental Design, Materials and Methods

### 4.1 Patient cohort

Initially, the dataset included images from 168 patients, of which 26 were excluded due to poor ultrasound image quality or a histological diagnosis other than glioma, resulting in a final cohort of 142 patients. The 142 subjects are patients who underwent brain tumor surgery between 2018 and 2023 in the centers previously detailed (RHUH, TMC, INCC, UPALER, CHRUT and MGH). Only patients diagnosed with glioma are included according to 2021 WHO classification of Central Nervous System tumors [19]. Table 2 provides a description of the clinical and radiological variables from each center and entire sample. Most of the gliomas included are grade 4; only CHRUT encompasses cases of lower grades as well. The IDH status—whether mutated or wild-type— was determined using immunohistochemistry techniques. MGMT methylation status is also presented in the cases available.

**Table 2:** Clinical and demographic sample features.

Variable	Centers						Total
	RHUH	TMC	CHRUT	INCC	UPALER	MGH	
**Total of subjects**	58	35	29	10	5	5	142
**Mean Age**	62.53± 10.82	46.34 ± 10.75	48.16 ± 14.16	58.9 ±19.78	67.2± 15.89	NA	55.88±14.45
**Sex**							
Male	35 (60.34%)	23 (65.71%)	9 (31.03%)	6 (60%)	3 (60%)	-	76 (53.52%)
Female	23 (39.66%)	12 (34.29%)	9 (31.03%)	4 (40%)	2 (40%)	-	50 (35.21%)
NA	-	-	11(37.93%)	-	-	5 (100%)	16 (11.27%)
**WHO grade**							
2	-	-	8 (27.59%)		-	-	8 (5.63%)
3	-	-	8 (27.59%)		-	-	8 (5.63%)
4	58 (100%)	35 (100%)	13 (44.83%)	10 (100%)	-	-	116 (81.69%)
NA	-	-	-	-	5 (100%)	5 (100%)	10 (7.04%)
**IDH status**							
Mutant	20 (34.48%)	6 (17.14%)	9 (31.03%)	-	-	-	35 (24.65%)
Wild-type	36 (32.07%)	29 (82.86%)	8 (27.59%)	10 (100%)	-	-	83 (58.45%)
NA	2 (3.45%)	-	12 (41.38%)	-	5 (100%)	5 (100%)	24 (16.90%)
**MGMT methylated**							
Yes	-	25 (71.43%)	11 (37.93%)	6 (60%)	-	-	42 (29.58%)
No	-	6 (17.14%)	2 (6.89%)	4 (40%)	-	-	12 (8.45%)
NA	58 (100%)	4 (11.43%)	16 (55.17%)		5 (100%)	5 (100%)	88 (61.97%)

The values are expressed as standard deviation and percentages as applicable. WHO = World Health Organization. IDH = isocitrate dehydrogenase. MGMT= O6-metilguanina-ADN-metiltransferasa

### 4.2 Imaging data

The dataset includes 1669 B-Mode ioUS images, all of them acquired previous surgical resection during craniotomy and cerebral tumor resection procedures. ioUS series were acquired using a sterilizable neuro-cranial transducer or probes covered with sterile sheets on a cart-based ultrasound system, depending on the manufacturer. The variability among the different ultrasound manufacturers, probe types, and frequencies is shown in Table 3.

**Table 3:** ioUS acquisition characteristics by center.

Variable	Centers						Total
	RHUH	TMC	CHRUT	INCC	UPALER	MGH	
**US manufacturer**	Hitachi	BK / Sonowand	Supersonic	Esaote	Esaote	BK	
**Type of probe**	Curved	Curved	Linear	Linear	Linear	Curved	
**Frequency**	4-8 Mhz	3 -8/5-13Mhz	4 – 15 Mhz	3 – 11 Mhz	3 – 11 Mhz	5 – 13 Mhz	
**Acquisition type**							
2D	58 (100%)	23 (65.72%)	29 (100%)	10 (100%)	5 (100%)	5 (100%)	118 (83.09%)
3D	-	12 (34.28%)	-	-	-	-	24 (16.90%)
**Number of images**	738	666	177	55	14	19	1669
**Median images per subject**	12 (7.75)	2 (34.0)	6 (4.0)	6 (2.5)	3 (1.0)	4 (2.0)	7 (10)

Values presented in parentheses correspond to interquartile ranges and percentages, as applicable. US = ultrasound. MHz = megahertz

The imaging plane was chosen to be as close as possible to orthogonal axes of the head (axial, sagittal, coronal), nevertheless this was often limited by the shape, size and location of the craniotomy. Images with suboptimal quality or artifacts that impeded processing and interpretation were excluded. All images available are in 2D format. The dataset includes 12 patients who registered as 3D ioUS volumes which are distinct from native 2D acquisition volumes. 3D images were decomposed into individual 2D axial slices, from which the most relevant slices were subsequently chosen (622images). The rest of the dataset was acquired in native 2D.

**Fig. 2:**
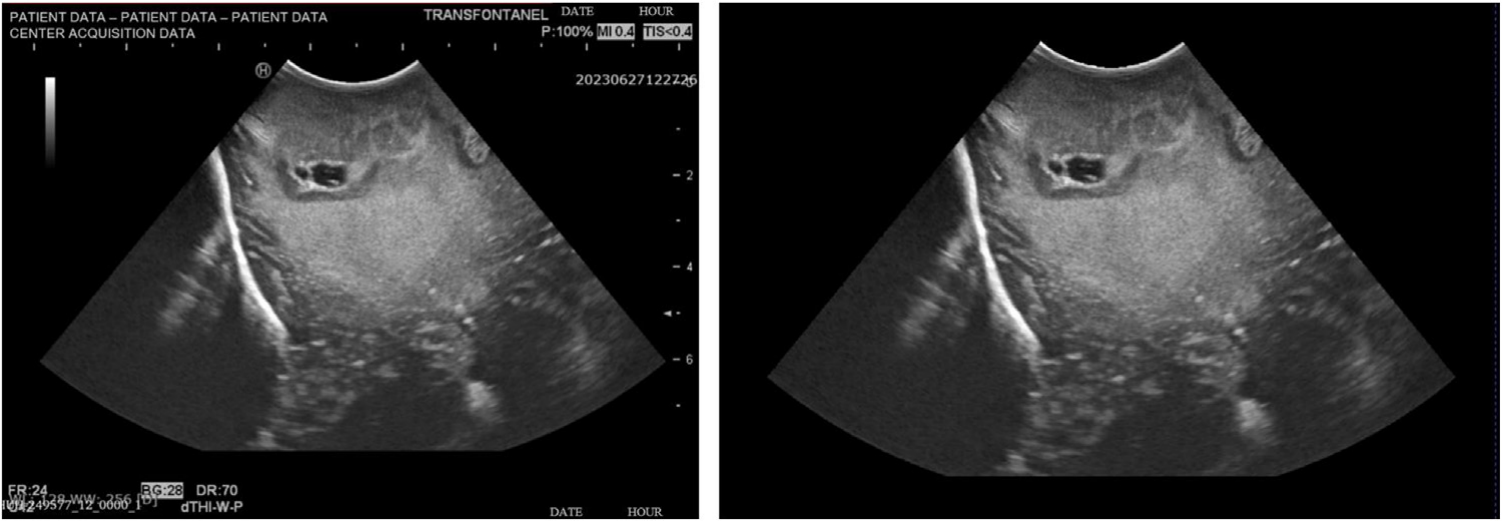
Two ioUS images pre (left) and post (right) being through anonymization automatic model. Identifying elements from the acquisition were removed from the images, ensuring that their integrity remained unaffected.

The images are provided in fully de-identified NIfTI (Neuroimaging Informatics Technology Initiative) format. Although the collected images lacked sensitive data and could be considered pseudo-anonymized, many of them contained acquisition elements such as dates, the name of the center, and other manufacturer-specific metadata. To ensure subject anonymization and data protection, a nnU-Net–based segmentation model was trained to identify and isolate the relevant content within the native field of view (FOV) (Fig. 2). nnU-Net [20] is a self-configuring deep learning framework commonly used for biomedical image segmentation. This process enables standardization and anonymization of the dataset, making it suitable for public release. The model was trained using 142 manually segmented images (one per subject). The manual segmentation was performed by a neurosurgeon specialized in interpretation of intraoperative images. Segmentation was made using 3D-Slicer via OpenIGTLink (Version 5.8.0). Validation of the model got a 0.98 Dice score. The model was applied to the rest of images from the dataset for anonymization, creating new images devoid of data or identifying information.

The mean image size is 640,728 pixels (SD = 861,051). Regarding the dimensions (width × height), 41.9% of the sample corresponds to images with dimensions of 800 × 900 pixels. The mean dimension is 756 × 583 pixels (IQR 367–800 pixels in width and 308–600 pixels in height). The variability observed in image dimensions arises from the heterogeneity of the sample in terms of acquisition protocols and imaging equipment. The minimum and maximum dimensions observed were 273 to 2,160 pixels in width and 275–1,398 pixels in height.

### 4.3 Tumor segmentation

**Fig. 3:**
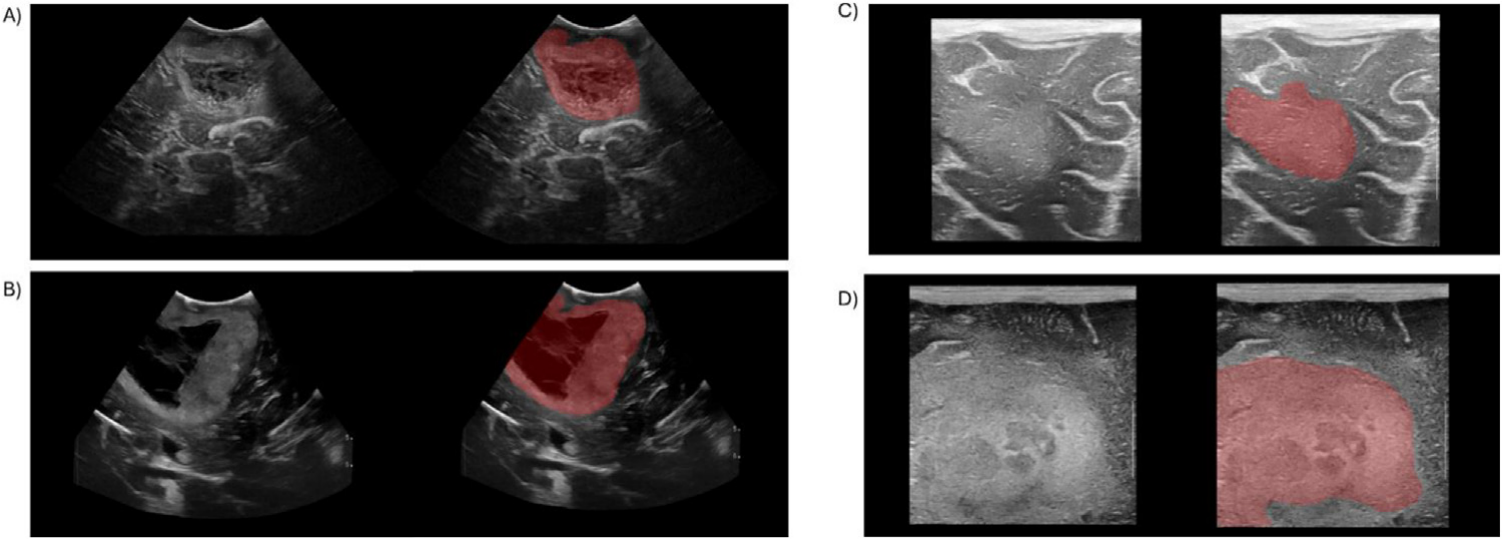
They are represented here different examples of ioUS images from the dataset and corresponding tumor mask. In A) and B) histological study was glioblastomas (IDH wild-type gliomas grade 4 CNS WHO classification), while the others are low grades: C) is an astrocytoma grade 2 and D) is an astrocytoma grade 3.

The dataset comprises tumor segmentations for all 1669 images, generated through automated pipeline based on the nnU-Net framework. This segmentation model is comprehensively described in the study conducted by Cepeda [2], where the BraTioUS dataset was utilized for both training and testing. The pseudolabels generated by this model were thoroughly reviewed and, when necessary, corrected by S.C., a neurosurgeon with 12 years of experience in medical imaging, particularly in the interpretation and analysis of intraoperative ultrasound. For this precision task, the ITK-SNAP software (Version 4.0.1, http://itksnap.org, accessed on 1 November 2024) was utilized to ensure true and reproducible segmentation. Consequently, the dataset consists of 1669 brain tumor segmentation masks of high accuracy, which are publicly available for unrestricted use for training deep learning models (Fig. 3).

**Fig. 4:**
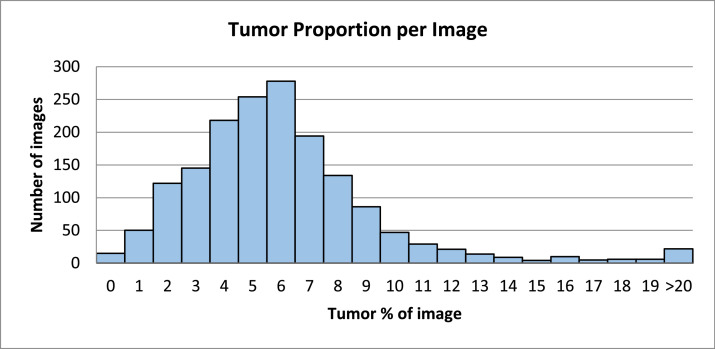
Histogram shows distribution of tumor size proportion per image. It should be noted that in most images, the size of the tumor mask is small relative to the overall image size (< 10% of iUS image size).

The average tumor size, measured in pixels, is 43,897 (SD= 77,645) with a minimum of 1,746 pixels and a maximum of 972,284 pixels. Fig. 4 illustrates the distribution of tumor sizes and their relationship to the overall image dimensions.

## Limitations

A notable constrain of the BraTioUS dataset is the absence of associated MRI scans of the patients, whether preoperative or intraoperative, which are available in the other previously mentioned datasets*.*

Another limitation is that the dataset comprises exclusively images obtained prior to the initiation of tumor resection, thereby lacking intraoperative acquisitions during or upon completion of the procedure. Consequently, except in reoperation cases, intraoperative ultrasound images do not encompass features such as the surgical cavity or residual tumor tissue.

## Ethics Statement

The project was conducted in accordance with the Declaration of Helsinki and approved by the Research Ethics Committee (CEIm) at Río Hortega University Hospital, Valladolid, Spain (Approval number 21-PI085. Date 30 April 2021). Informed consent was obtained from all subjects involved in the study.

The authors have read and follow the ethical requirements for publication in Data in Brief and confirming that the current work does not involve human subjects, animal experiments, or any data collected from social media platforms.

## CRediT Author Statement

**Olga Esteban-Sinovas:** Methodology, Investigation, Resources, Data Curation, Writing – Original Draft, Visualization, Validation; **Rosario Sarabia:** Writing – Review & Editing, Investigation, Resources; **Ignacio Arrese:** Writing – Review & Editing, Investigation, Resources; **Vikas Singh:** Investigation, Resources; **Prakash Shetty:** Investigation, Resources, **Aliasgar Moiyadi:** Investigation, Resources; **Ilyess Zemmoura:** Investigation, Resources; **Massimiliano Del Bene:** Investigation, Resources; **Arianna Barbotti:** Investigation, Resources; **Francesco DiMeco:** Investigation, Resources; **Timothy Richard West:** Investigation, Resources; **Brian Vala Nahed:** Investigation, Resources; **Giuseppe Roberto Giammalva:** Investigation, Resources; **Santiago Cepeda:** Conceptualization, Supervision, Software, Project Administration, Writing – Review & Editing.

## Data Availability

ZENODOBraTioUS - Brain Tumor Intraoperative Ultrasound Dataset (Original data).
